# Editorial: Recruitment of leukocytes during resolution of inflammation

**DOI:** 10.3389/fimmu.2026.1808267

**Published:** 2026-03-03

**Authors:** Vivian Louise Soares Oliveira, Sofie Struyf

**Affiliations:** Laboratory of Molecular Immunology, Department of Microbiology, Immunology and Transplantation, Rega Institute, KU Leuven, Leuven, Belgium

**Keywords:** cell recruitment, immune cell migration, leukocytes, pro-resolution mediators, resolution of inflammation

Leukocyte recruitment is traditionally viewed as a hallmark of inflammation initiation and propagation, enabling rapid host defense. Equally important, however, is the precise regulation of this process, including its timing, magnitude, localization, and termination, which ultimately determines whether inflammation resolves or progresses toward chronic pathology (1–3). Classically, resolution of inflammation was considered a passive process coinciding with dissipation of inflammatory mediators and a halt in immune cell influx. Accumulating evidence now challenges this oversimplified view, revealing resolution as an active and highly coordinated process that depends not on immune silence, but on precisely regulated leukocyte recruitment, functional reprogramming, and clearance (4, 5). Thus, leukocyte recruitment is not simply switched off, but reshaped and redirected, and involves regulation of cell fate and tissue restoration. The articles gathered in this Research Topic collectively reflect this expanded view across inflammatory contexts, disease states, and therapeutic interventions.

To address how leukocyte migration acts as a central coordinator of inflammatory responses and immune surveillance, Song et al. contribute to this Research Topic with a review of the tightly regulated, multistep trafficking of diverse immune cell populations to inflamed tissues. This review integrates the molecular mechanisms governing these processes with current and emerging therapeutic strategies and technologies, highlighting both the translational potential and the unresolved challenges associated with targeting immune cell migration in inflammatory diseases. The authors also describe successful clinical interventions targeting leukocyte migration in conditions such as multiple sclerosis (MS) and inflammatory bowel disease (IBD), while emphasizing the need for more selective, context-specific, and clinically translatable therapeutic approaches to fully realize the potential of migration-targeted immunotherapy.

Modulation of the chemokine system represents another major strategy for targeting leukocyte recruitment (6–8). Chemokines orchestrate immune cell trafficking but also regulate retention, positioning, and functional activation. As reviewed by Oliveira et al. in this Research Topic, chemokine functions extend beyond inflammation initiation. During resolution, changes in chemokine and chemokine receptor expression reduce inflammatory leukocyte numbers at inflamed sites while enabling the recruitment and polarization of regulatory, reparative, or pro-resolving cells. Conversely, persistent chemokine signaling can sustain inflammatory infiltrates and prevent proper resolution. Given the complexity and redundancy of the chemokine network, therapeutic strategies must be precisely timed and spatially controlled to avoid disrupting essential resolution pathways while attempting to suppress inflammation. Nevertheless, targeting specific components of the chemokine system to modulate inflammation and actively promote resolution remains a promising avenue for future investigations.

Resolution of inflammation requires not only the cessation of leukocyte recruitment, but also the controlled removal of immune cells that have already fulfilled their effector functions. Neutrophils, in particular, must undergo timely apoptosis followed by efficient clearance to prevent collateral tissue damage (9, 10). Specialized pro-resolving mediators (SPMs) have emerged as critical regulators of this process, actively promoting neutrophil apoptosis while supporting macrophage-mediated efferocytosis and tissue repair (11, 12). As discussed by Kang et al. in this Research Topic, unresolved inflammation characterized by the high numbers of neutrophils can lead to progressive tissue injury in kidney diseases. Thus, induction of neutrophil apoptosis represents a promising therapeutic strategy. The authors provide mechanistic insight into how resolution is actively enforced at the cellular level, demonstrating that SPM-driven neutrophil apoptosis and clearance limit tissue damage while fostering a microenvironment conducive to repair and regeneration in the kidney.

Importantly, reducing neutrophil numbers is not universally beneficial. Hernandez et al. demonstrate that partial depletion of circulating neutrophils in a murine model of LPS-induced severe systemic inflammation exacerbates inflammatory responses and worsens clinical outcomes, most likely through mechanisms involving interleukin (IL)-10. These findings challenge the assumption that limiting neutrophil availability will invariably attenuate inflammation. Instead, these findings support a more nuanced perspective whereby neutrophils participate not only in amplifying inflammation but also in promoting host protection and, potentially, inflammatory resolution. Together, these observations reinforce the concept that the timing, context, and extent of leukocyte recruitment critically shape inflammatory outcomes.

The complexity of therapeutically manipulating leukocyte recruitment is further illustrated in the context of cancer. Granulocyte colony-stimulating factor (G-CSF) is widely used to prevent neutropenia in patients undergoing chemotherapy. However, as reviewed by Krykbaeva et al. in this Research Topic, G-CSF administration may have additional and unintended consequences in patients receiving chemo-immunotherapy with immune checkpoint inhibitors. While enhanced neutrophil survival and recruitment can support immune competence and treatment tolerance, in preclinical models, G-CSF seemed to promote neutrophil polarization toward a pro-tumorigenic phenotype under certain conditions, negatively affecting the tumor microenvironment and contributing to tumor growth and metastasis. The authors highlight key mechanistic insights, emerging clinical signals, and existing gaps in evidence, ultimately emphasizing the importance of adhering to strict, consensus-based guidelines for G-CSF use in the context of chemo-immunotherapy. From a resolution perspective, these findings raise important questions about how artificially modulated leukocyte recruitment intersects with endogenous regulatory pathways and may yield unexpected outcomes.

Beyond systemic inflammation, aberrant leukocyte recruitment also underlies tissue-specific autoimmune diseases. In alopecia areata (AA), chemokine-driven immune cell recruitment to the hair follicles leads to localized tissue damage and chronic inflammation. In this Research Topic, Van Caelenberg et al. analyze chemokine profiles in AA through a meta-analysis, identifying elevated Th1-associated chemokines, a distinct Th2 signature, and chemokines linked to monocyte, dendritic cell, and eosinophil recruitment. The findings suggest the involvement of immune cells beyond classical T helper subsets and illustrate how sustained recruitment can prevent immune homeostasis.

By bringing together studies encompassing molecular mediators, experimental models, clinical interventions, and tissue-specific diseases, this Research Topic underscores that leukocyte recruitment is not a discrete event confined to the early stages of inflammation, but rather a dynamic process that both influences and is influenced by resolution mechanisms. Recruitment determines which leukocytes arrive, how long they are retained, how they interact with the tissue microenvironment, and ultimately whether inflammation resolves or progresses to chronicity. Rather than simply blocking or indiscriminately enhancing leukocyte migration, future therapeutic strategies should aim to guide this process in a context-dependent manner. Advances in spatial and single-cell technologies now offer unprecedented opportunities to map leukocyte recruitment pathways with high temporal and anatomical resolution, enabling the identification of biomarkers that distinguish inflammatory from pro-resolving immune responses. A deeper understanding of the immune system’s mechanisms for resolution may ultimately support more precise and durable control of inflammation, while preserving host defense and tissue function.
